# Effects of Feeding Methionine Hydroxyl Analogue Chelated Zinc, Copper, and Manganese on Growth Performance, Nutrient Digestibility, Mineral Excretion, and Welfare Conditions of Broiler Chickens: Part 2: Sustainability and Welfare Aspects

**DOI:** 10.3390/ani15030419

**Published:** 2025-02-03

**Authors:** Hoang Duy Nguyen, Amy Fay Moss, Frances Yan, Hugo Romero-Sanchez, Thi Hiep Dao

**Affiliations:** 1 School of Environmental and Rural Science, Faculty of Science, Agriculture, Business and Law, University of New England, Armidale, NSW 2351, Australia; hnguye66@myune.edu.au (H.D.N.); amoss22@une.edu.au (A.F.M.); 2 Novus International, Inc., 20 Research Park Drive, St. Charles, MI 63304, USA; frances.yan@novusint.com (F.Y.); hugo.romero@novusint.com (H.R.-S.); 3 Faculty of Animal Science, Vietnam National University of Agriculture, Trau Quy Town, Gia Lam District, Hanoi 100000, Vietnam

**Keywords:** trace minerals, organic mineral, meat chicken, sustainability, environment

## Abstract

This study investigated the effects of the dietary supplementation of mineral methionine hydroxyl analogue chelates (MMHACs) zinc (Zn), copper (Cu), and manganese (Mn) on excreta nitrogen and mineral levels, housing conditions, and the welfare status of broilers. Three-hundred eighty-four day-old Ross 308 male chicks were randomly distributed to four dietary treatments, each consisting of eight replicate pens of twelve birds per pen. The treatments were (1) inorganic trace mineral ZnSO_4_ (110 ppm), CuSO_4_ (16 ppm), and MnO (120 ppm) (ITM); (2) MMHAC Zn (40 ppm), Cu (10 ppm), and Mn (40 ppm) (M10); (3) inorganic trace mineral ZnSO_4_ (110 ppm), tribasic copper chloride (125 ppm), and MnO (120 ppm) (T125); and (4) MMHAC Zn (40 ppm), Cu (30 ppm), and Mn (40 ppm) (M30). Three feeding phases including the starter (days 0–10), grower (days 10–21), and finisher (days 21–42) were used. The findings showed that birds offered MMHACs at both levels had significantly lower Zn and Mn levels, and birds offered the T125 diet had higher Cu levels in the excreta compared to those fed the other diets on days 10, 16, 21, 28, and 42 (*p* < 0.001). The life cycle assessment showed that MMHAC supplementation at 30 ppm can be expected to improve the sustainability of the poultry industry in terms of reduced emissions into the environment, whereas excreta nitrogen and moisture content, litter conditions, levels of air gases (ammonia, carbon dioxide, and methane), and welfare indicators were similar between the dietary treatments. Hence, the supplementation of MMHACs to broiler diets at 30 ppm could maintain litter quality and welfare status while reducing emissions into the environment and the Zn, Mn, and/or Cu excretion of broilers, therefore reducing the environmental impacts of broiler production.

## Simple Summary

Due to the convenience, reasonable prices, and probably the lack of data on precise mineral requirements, inorganic trace minerals including zinc, copper, and manganese are typically used at high levels in broiler diets to compensate for the low bioavailability of this mineral source. However, a high intake of inorganic trace minerals usually causes high mineral excretion in the excreta, which is potentially harmful to the environment. This study investigated the effects of the dietary supplementation of chelated trace minerals on air gas levels in the broiler shed, welfare conditions, the excreta mineral and nitrogen content of broilers and further explored the environmental impact via a life cycle assessment. The findings of this study may help to improve the sustainability of the poultry industry in terms of reduced emissions into the environment.

## 1. Introduction

Broiler meat is widely recognized as the most cost-effective and sustainable source of animal protein, primarily due to the remarkable efficiency of broiler chickens in converting feed into meat [1]. Zinc (Zn), copper (Cu), and manganese (Mn) are important trace minerals playing vital roles in metabolism and growth, thereby influencing the productivity and general health conditions of broiler chickens [2]. Due to the convenience, reasonable prices, and probably the lack of data on precise mineral requirements, inorganic trace minerals including Zn, Cu, and Mn are typically used at high levels in the broiler diets to compensate for the low bioavailability of this mineral source [3,4]. However, according to Bhagwat et al. [5], two main disadvantages may arise from using inorganic trace minerals in broiler diets. Firstly, Cu sulphate and Zn oxide obtained from the metal industry are typically used in poultry diets; however, this source of Cu and Zn is usually contaminated with fluorine and cadmium [5,6]. Secondly, the metabolism and absorption of the minerals may be decreased due to the antagonisms among inorganic mineral sources [5]. In addition, the high intake of inorganic trace minerals is not only wasteful but also potentially harmful to the ecosystem, as most inorganic trace minerals are excreted as waste, and the body stores them only to a very small extent [7]. In this regard, organic trace minerals are acknowledged to be a more readily available source of trace elements compared to conventional inorganic alternatives like sulphates or oxides [8], reducing mineral content in litter [9]. In contrast to inorganic mineral salts, which bind metal ions through electrovalency, organic trace minerals are coordination compounds that form metal complexes [2]. Previous studies have shown that feeding organic trace minerals at 30 to 50% of the inorganic trace mineral levels did not affect growth performance while significantly lowering Mn and Zn excretion in broilers [2,10].

Excess mineral excretion, particularly heavy metals, due to the high levels and low bioavailability of inorganic trace minerals in conventional broiler diets has raised environmental concerns [11]. The accumulation of excess minerals in the litter and soil may not only contaminate soil and surface water but also reduce crop yield [12,13]. According to Dozier III et al. [14], poultry manure contains 660% and 560% higher amounts of Zn and Cu, respectively, than that required by crops. Therefore, sustainable broiler production has faced various challenges including improving birds’ productivity, minimizing environmental impacts, ensuring economic feasibility, and upholding social responsibility [15,16]. Life cycle assessment has been recognized as an effective tool for assessing environmental aspects of poultry production [17]. In more detail, the life cycle assessment examines environmental aspects, including resource utilization and the environmental consequences of activities occurring throughout a product’s entire life cycle [18]. Hence, the life cycle assessment is well suited for environmental assessment and has been employed widely to evaluate the environmental impacts of broiler production [19]. However, there are no studies investigating the effects of organic mineral supplementation on environmental aspects in broiler production using the life cycle assessment.

Promoting birds’ welfare is crucial to improve the sustainability of the broiler industry [20]; however, information regarding the effects of feeding organic trace minerals or high dietary Cu levels on welfare indicators in broiler chickens is limited. It has been suggested that hock joint conformity, an important factor controlling birds’ walking ability, is less sensitive to Zn deficiency than Mn and that a higher Mn level is required to support normal joint conformity than growth [21]. Copper also plays certain roles in maintaining tissue structural integrity as it serves as a cofactor for the lysyl oxidase, an enzyme participating in the cross-linking of elastin proteins and collagen [22]. Kim et al. [23] reported decreased skin tearing and increased skin collagen content in broilers supplemented with organic Zn. Additionally, Zhao et al. [4] observed the tendency of lower footpad lesion scores in birds fed diets supplemented with 40 ppm of Zn, 60 ppm of Mn, and 8 ppm of Cu in chelated forms (Mintrex) and 40 ppm of Zn, 60 ppm of Mn and 8 ppm of Cu in inorganic forms (sulphates) compared to birds offered inorganic trace minerals (sulphates) with 80 ppm of Zn, 120 ppm of Mn, and 8 ppm of Cu. Hence, replacing inorganic trace minerals with organic alternatives may help to improve the welfare standard and health conditions of broiler chickens.

The existing literature evidence suggests that the replacement of inorganic with organic trace minerals may improve the sustainability of the broiler industry by generating numerous welfare and environmental benefits. This study was conducted to investigate the effects of supplementing mineral methionine hydroxyl analogue chelates (MMHACs) Zn, Cu, and Mn (MINTREX^®^ Zn, Cu, and Mn trace minerals, Novus International, Inc., St. Charles, MI, USA) at recommended rates (40, 10, and 40 ppm of Zn, Cu, and Mn, respectively) in a traditional broiler diet on the sustainability aspects including excreta nitrogen and mineral levels, housing conditions, and the welfare status of broilers. Additionally, further treatments explored the environmental and welfare impacts of Cu as a growth promoter under the same conditions using either an inorganic Cu salt (125 ppm) or a reduced rate of Cu chelate (30 ppm). This paper is the second part in the series investigating the effects of feeding MMHACs and high dietary inorganic trace mineral levels in broiler chickens. The results on growth performance, carcass weight and quality, nutrient digestibility, gizzard erosion score, and bone parameters were reported by Nguyen et al. [24]. The results showed that the supplementation of MMHACs to broiler diets at 30 ppm possibly increased body weight gain and feed intake compared to the control inorganic trace mineral diet [24]. Furthermore, feeding MMHACs at 30 ppm significantly increased the thigh and drumstick processing weight and Cu digestibility while maintaining the bone health of broilers [24].

## 2. Materials and Methods

This research was conducted at the Centre of Animal Research and Teaching at the University of New England (UNE), Armidale, New South Wales, Australia. Approval for this study was granted by the UNE Animal Ethics Committee under approval number ARA23-004, ensuring compliance with the guidelines described in the Australian code of practice for the ethical use and care of animals in scientific research [25].

### 2.1. Experimental Design and Diets

The experimental broiler shed was fully enclosed and equipped with tunnel ventilation, allowing precise control over temperature and humidity levels. Throughout the initial brooding phase (days 0–10), indoor temperature was maintained using three electric brooders with thermostatic controls. Thirty-two broiler floor pens were used, each containing a manual feeder and two nipple drinkers, with an approximate area of 1 m^2^ per pen, excluding space occupied by feeders and drinkers. A total of 384-day-old Ross 308 male chicks were randomly allocated to four dietary treatments, each comprising eight replicate pens with 12 chicks per pen per treatment. The starting weights of the pens were comparable across all treatments, with no significant differences observed (*p* > 0.05). Chicks were raised on hardwood shavings as bedding material within an environmentally controlled room over a 42-day feeding trial. All birds received feed and water within 12 h of hatching and were vaccinated against infectious bronchitis virus (IBV) and Newcastle disease virus (NDV) using a combination vaccine (1 × IBV/NDV vaccine) according to the hatchery’s vaccination protocol (Aviagen Chicken Hatchery, Goulburn, New South Wales, Australia). Throughout this study, birds had ad libitum access to the feed and water. The lighting, temperature, and ventilation conditions inside the broiler shed adhered to the recommendations outlined for Ross 308 broilers [26]. Birds were monitored twice daily to ensure their general health and the continuous availability of feed and water. The feed was pelleted at 65 °C and was provided in crumble form during the starter phase (days 0–10) and pellets during the grower phase (days 10–21) and the finisher phase (days 21–42). Four treatments including (1) inorganic trace mineral ZnSO_4_ (110 ppm), CuSO_4_ (16 ppm), and MnO (120 ppm) (ITM); (2) MMHAC Zn (40 ppm), Cu (10 ppm), and Mn (40 ppm) (M10); (3) inorganic trace mineral ZnSO_4_ (110 ppm), tribasic copper chloride (125 ppm), and MnO (120 ppm) (T125); and (4) MMHAC Zn (40 ppm), Cu (30 ppm), and Mn (40 ppm) (M30) were used in this study. Levels of Zn, Cu, and Mn in the ITM treatment followed the Ross 308 nutritional recommendation [27], while levels of Zn, Cu, and Mn in the M10, M30, and T125 treatments were selected based on previously published reports [28,29,30]. The vitamin–mineral premix with desirable Zn, Cu, and Mn levels and sources for each treatment were produced and provided by an experienced manufacturer (Rabar Pty Ltd., Beaudesert, Queensland, Australia). Supplemental levels of crystalline methionine in the M10 and M30 diets were decreased to ensure similar methionine levels across treatments. Wheat served as the filler in all diets, with titanium dioxide added to the grower diets at 0.5% as an inert dietary marker for nutrient digestibility determination.

Prior to the diet formulation, the nutrient content of soybean meal, sorghum, and wheat was measured using near-infrared reflectance spectroscopy (Foss NIR 6500, Hillerød, Denmark) standardized with Adisseo calibration. Additionally, the crude protein content of major feed ingredients was analyzed using a nitrogen analyzer (LECO Corporation, St Joseph, MI, USA) with EDTA as a calibration standard prior to diet formulation. Diet formulation was conducted using a commercial feed formulation software (Concept 5, CFC Tech Services, Inc., Browerville, MN, USA), ensuring that dietary nutrient levels of all treatments met Ross broiler nutrition specifications [27]. Standard methods [31] were used to determine gross energy, crude protein, dry matter, and mineral levels of the mixed diets to confirm formulated levels. Detailed information regarding diet composition and nutrient content is given in Appendix Table A1 and Table A2.

### 2.2. Data Collection

#### 2.2.1. Excreta Mineral, Nitrogen, and Moisture Content

On days 10, 16, 21, 28, 35, and 42, representative fresh excreta samples were collected from each pen into 70 mL containers for the analysis of moisture content, nitrogen, and minerals. Subsequently, the collected excreta samples were thoroughly mixed and sub-sampled for further analysis. A small portion was aliquoted to determine the moisture content, employing a forced air oven around 48 h at 105 °C until constant weights were achieved. The remainder were stored at −20 °C for subsequent measurements. Excreta samples were freeze-dried until constant weights using a freeze dryer machine (Christ Alpha 1-4 LDplus, Osterode am Harz, Germany). Subsequently, the dried excreta samples were ground finely to particle sizes of 0.5 mm. Finally, the ground dried excreta samples were measured for the nitrogen and mineral content using a nitrogen analyzer instrument (LECO Corporation, St Joseph, MI, USA) calibrated with EDTA and the inductively coupled plasma-optical emission spectrometry machine (Agilent, Mulgrave Australia), respectively.

#### 2.2.2. Measurement of Environmental Conditions

On days 21 and 42, litter scoring was performed from 5 different areas in each pen (4 corners and middle of the pens) following the Welfare Quality^®^ assessment protocol for poultry [32]. Specifically, the scoring scores were 0 for litter that was entirely dry and flaky; 1 for litter that was dry but not easily moved with a boot; 2 for litter that left a foot imprint and could be shaped into a ball that readily falls apart; 3 for litter that was adhered to boots and could be shaped into a firm ball; and 4 for litter that was wet and adhesive under a hard crust. Furthermore, litter samples were collected from each pen (5 different locations including 4 corners and the middle of the pens) for the measurement of litter moisture content on days 21 and 42. Litter samples were then mixed thoroughly, and sub-samples were removed for the determination of moisture content by the forced air oven for approximately 48 h at 105 °C (to constant weight).

On days 21 and 42, the concentrations of carbon dioxide (CO_2_), methane (CH_4_), and ammonia (NH_3_) in each pen were assessed using a CO_2_ metre (IC-CENTER512 CO2 Meter, Synotronics Pty Ltd., Dry Creek, South Australia, Australia), CH_4_ metre (Series 300 Lithium Portable Air Quality Monitor (IC-HH-S300L) with CH_4_ Sensor Head (IC-SH-MT), Synotronics Pty Ltd., Dry Creek, South Australia, Australia), and ammonia metre (GasAlert Extreme NH3 (GAXT-A2-DL), Gas-Sensing, Inwood, IA, USA). The devices were calibrated to ensure accurate measurements of the target gases. The gas levels were recorded at 5 different locations within each pen, including 4 corners and the middle of the pens, at a height of approximately 20 cm above the litter surface.

#### 2.2.3. Life Cycle Assessment

The Poultry Greenhouse Accounting Framework V1.45 from the Primary Industries Climate Change Centre [33] was utilized to determine the emissions (CO_2_; CH_4_; nitrous oxide (N_2_O)), the total t CO2e/farm, and the total t CO2e/farm per kilo live weight gain in a simulation of a total of 320,000 broiler chickens across 3 grow-out periods of 42 days each. The following data were included in the calculator for each dietary treatment; birds’ live body weight, diet composition, dry matter intake and total feed consumption, dry matter digestibility measured by the titanium dioxide marker as described in Nguyen et al. [24] (72.76%, 74.63%, 74.13%, and 73.68% for ITM, M10, T125 and M30 treatments, respectively), excreta crude protein and ash content, and the N retention rate.

#### 2.2.4. Welfare Indicators

Welfare indicators including foot pad lesions, hock burns, plumage cleanliness, leg deformity, and walking ability were assessed on 3 birds per pen on days 21 and 42 following Royal Society for the Protection of Cruelty to Animals (RSPCA) guidelines [34].

### 2.3. Statistical Analysis

The obtained data were analyzed using the R Commander (version 3.3.1, R Foundation for Statistical Computing, Vienna, Austria) to test statistical differences among the dietary treatments. Initial data examination involved visualization via scatterplots, boxplots, histograms, and the assessment of data normality using the Shapiro–Wilk test. Depending on the results of the data normality test and homogeneity of variances between the dietary treatments (Levene’s test), either a one-way ANOVA or non-parametric ANOVA (Kruskal–Wallis test) was applied to test statistical differences among the dietary treatments. Tukey’s post hoc test was utilized for mean separation following a significant ANOVA result. Data on litter scores and welfare indicators including foot pad lesions, hock burns, plumage cleanliness, leg deformity, and walking ability scoring on days 21 and 42 were not normally distributed and thus were analyzed by the Kruskal–Wallis test; however, no significant results were obtained for these variables. Significance was determined at a *p*-value ≤ 0.05, and a possible effect was noted for values falling within 0.05 < *p*-value < 0.10.

## 3. Results

### 3.1. Excreta Mineral, Nitrogen, and Moisture Content

**Figure 1:**
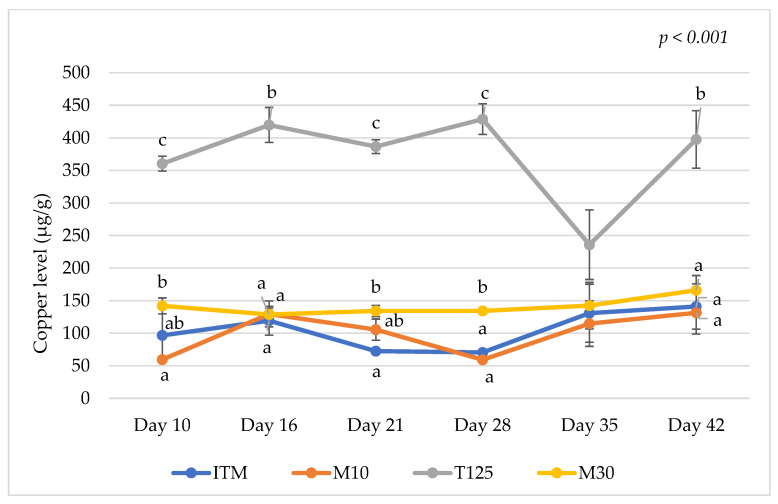
Copper levels in the excreta of the dietary treatments during this study. ITM: inorganic trace mineral ZnSO_4_ (110 ppm), CuSO_4_ (16 ppm), and MnO (120 ppm); M10: mineral methionine hydroxyl analogue chelates Zn (40 ppm), Cu (10 ppm), and Mn (40 ppm); T125: inorganic trace mineral ZnSO_4_ (110 ppm), tribasic copper chloride (125 ppm), and MnO (120 ppm); and M30: mineral methionine hydroxyl analogue chelates Zn (40 ppm), Cu (30 ppm), and Mn (40 ppm). Plot points marked with different letters (a–c) indicate significant differences between means at the same age based on the results of Tukey’s post hoc test (*p* < 0.05). Vertical lines over the plot points represent standard errors of the means.

**Figure 2:**
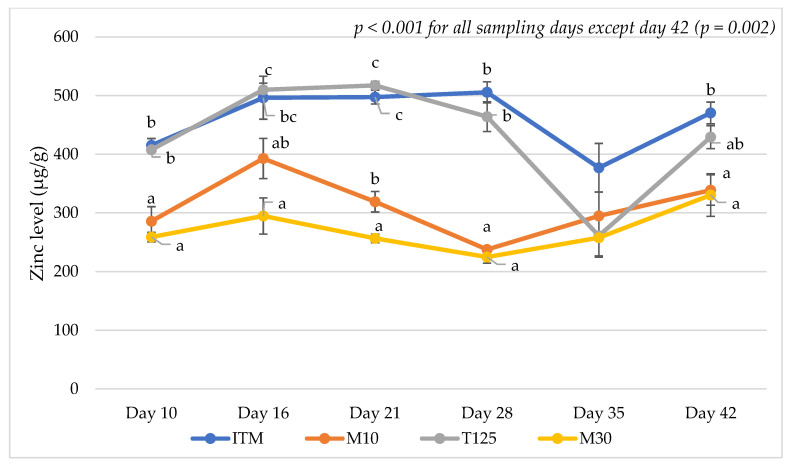
Zinc levels in the excreta samples of the dietary treatments during this study. ITM: inorganic trace mineral ZnSO_4_ (110 ppm), CuSO_4_ (16 ppm), and MnO (120 ppm); M10: mineral methionine hydroxyl analogue chelates Zn (40 ppm), Cu (10 ppm), and Mn (40 ppm); T125: inorganic trace mineral ZnSO_4_ (110 ppm), tribasic copper chloride (125 ppm), and MnO (120 ppm); and M30: mineral methionine hydroxyl analogue chelates Zn (40 ppm), Cu (30 ppm), and Mn (40 ppm). Plot points marked with different letters (a–c) indicate significant differences between means at the same age based on the results of Tukey’s post hoc test (*p* < 0.05). Vertical lines over the plot points represent standard errors of the means.

**Figure 3:**
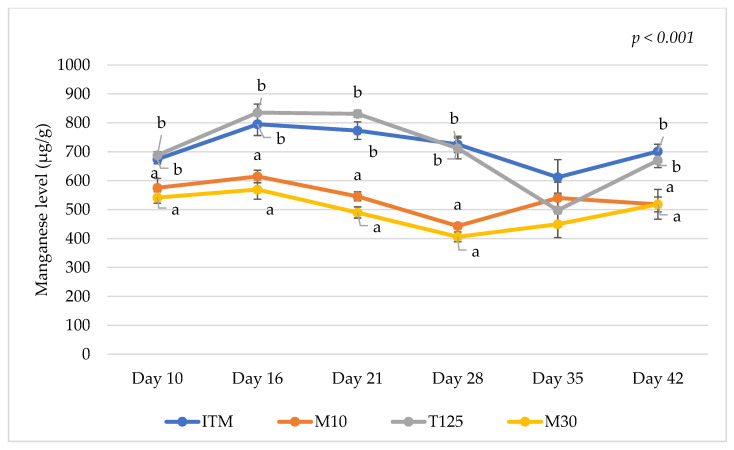
Manganese levels in the excreta samples of the dietary treatments during this study. ITM: inorganic trace mineral ZnSO_4_ (110 ppm), CuSO_4_ (16 ppm), and MnO (120 ppm); M10: mineral methionine hydroxyl analogue chelates Zn (40 ppm), Cu (10 ppm), and Mn (40 ppm); T125: inorganic trace mineral ZnSO_4_ (110 ppm), tribasic copper chloride (125 ppm), and MnO (120 ppm); and M30: mineral methionine hydroxyl analogue chelates Zn (40 ppm), Cu (30 ppm), and Mn (40 ppm). Plot points marked with different letters (a–b) indicate significant differences between means at the same age based on the results of Tukey’s post hoc test (*p* < 0.05). Vertical lines over the plot points represent standard errors of the means.

The analyzed nutrient content of the dietary treatments is shown in Appendix Table A2. The results on mineral levels in the excreta on days 10, 16, 21, 28, 35, and 42 are presented in Table 1, Table 2, Table 3, Table 4, Table 5 and Table 6, respectively. Additionally, Cu, Zn, and Mn levels in the excreta of the dietary treatments over the duration of this study are shown in Figure 1, Figure 2 and Figure 3, respectively. Birds offered the M10 and M30 diets had lower excreta Zn and Mn levels compared to those offered the T125 and ITM control diets on days 10, 16, 21, 28, and 42 (*p* < 0.001 for all sampling days except Zn level at day 42 (*p* = 0.002)). Birds fed the T125 diet exhibited higher excreta Cu levels compared to those fed the other diets on days 10, 16, 21, 28, and 42 (*p* < 0.001). Lowering Cu level sin the M10 diet resulted in lower excreta Cu levels on day 10 (*p* < 0.001, Table 1) and day 28 (*p* < 0.001, Table 4) but higher excreta Zn levels on day 21 (*p* < 0.001, Table 3) and higher excreta Ca levels on day 28 (*p* < 0.001, Table 4) compared to the M30 group. The results also showed that birds fed the T125, M10, and M30 diets had higher excreta Ca and Na levels with the highest excreta Ca and Na levels observed for the T125 group compared to the ITM group on day 10 (*p* < 0.001, Table 1). On day 28, higher excreta Ca (*p* < 0.001) and Na (*p* = 0.008) levels were observed in birds fed the ITM and M10 diets compared to those fed the T125 diet (Table 4). Similarly, higher excreta Ca levels were observed in birds fed the ITM and M10 diets compared to those fed the T125 diet on day 35 (*p* = 0.003, Table 5). On day 42, higher excreta Ca levels were observed in birds fed the M10 diet compared to birds fed the T125 diet (*p* = 0.037, Table 6).

Birds fed the M30 diet possibly had lower excreta Mg levels on day 16 (*p* = 0.060, Table 2) and lower excreta Mg levels on day 35 (*p* = 0.002, Table 5) compared to those fed the ITM diet. Birds fed the T125 diet also had lower excreta Mg levels compared to birds fed the ITM and M10 diets on day 35 (*p* = 0.002, Table 5). Excreta P levels were higher in birds fed the T125 diet compared to birds fed the M30 diet on day 21 (*p* = 0.05, Table 3). Additionally, higher excreta S levels were observed in birds fed the T125 and ITM diets compared to those fed the M30 diet on day 21 (*p* = 0.009, Table 3) and day 28 (*p* = 0.018, Table 4). Birds fed the ITM diet possibly had higher excreta Co levels compared to the M30 group on day 21 (*p* = 0.066, Table 3) and higher excreta Co levels compared to the M10 and M30 groups on day 28 (*p* = 0.001, Table 4) and the T125 and M30 groups on day 35 (*p* = 0.024, Table 5).

The results on nitrogen and moisture content in the excreta on days 10, 16, 21, 28, 35 and 42 are presented in Table 7 and Table 8, respectively. On day 10, birds offered the T125 diet had lower excreta nitrogen content compared to those offered the ITM diet (*p* < 0.001, Table 7). Additionally, birds fed the M10 diet had lower excreta nitrogen content compared to those fed the ITM diet on day 10 (*p* < 0.001, Table 7), whereas excreta moisture content was not different between the dietary treatments at all sampling days (Table 8).

### 3.2. Housing Conditions

The results on the litter score and moisture content on days 21 and 42 are shown in Table 8. Birds fed the ITM control diet possibly had higher litter scores compared to the other treatment groups on day 21 (*p* = 0.056). The litter moisture content on day 21 and litter score and moisture content on day 42 were not affected by the dietary treatments. The results on NH_3_, CO_2_, and CH_4_ levels in the pens are described in Table 9. The NH_3_ and CO_2_ levels on day 21 and NH_3_, CO_2_, and CH_4_ levels on day 42 were not different between the dietary treatments. Methane was not detected at all pens on day 21 possibly due to its extremely low level in the broiler shed at this age.

### 3.3. Life Cycle Assessment

The results on the life cycle assessment of the dietary treatments over the entire study are shown in Table 10. It was simulated that the M10 treatment had higher amounts of emissions from CO_2_, CH_4_, and N_2_O (fuel, manure management, atmospheric deposition, leaching, and runoff) compared to the ITM and M30 treatment (*p* = 0.007) and higher total t CO2e/farm compared to the M30 treatment (*p* = 0.013). Meanwhile, the T125 treatment possibly had higher simulated amounts of emissions from purchased feed, electricity, and fuel compared to the ITM treatment (*p* = 0.076) and higher total t CO2e/farm per kg live weight gain compared to the other treatments (*p* < 0.001). Additionally, it was simulated that the M30 treatment had the least amount of emissions from CO_2_, CH_4_, and N_2_O (*p* = 0.007) and the least total t CO2e/farm (*p* = 0.013), whereas the control ITM treatment had the least amount of emissions from purchased feed, electricity, and fuel (*p* = 0.076) due to the reduced feed intake of this treatment. Furthermore, due to the improved body weight gain, the lowest total t CO2e/farm per kg live weight gain was also achieved by the M30 treatment, which was significantly lower than that of the T125 treatment (*p* < 0.001).

The results on welfare indicators including foot pad lesions, hock burns, plumage cleanliness, leg deformity, and walking ability scoring on days 21 and 42 are presented in Table 11. Hock burns and leg deformity were not observed in any birds on day 21. Other welfare indicators were not different between the dietary treatments on both day 21 and day 42.

## 4. Discussion

Diet formulation with large safety margins on supplemental levels of inorganic trace minerals in broiler production causes excessive mineral excretion [3]. High levels of trace minerals such as Zn, Cu, Mn, and Fe in poultry litter are of concern as they may be toxic and reduce crop yield [12]. It is known that organic trace minerals have higher bioavailability compared to inorganic sources. Thus, organic trace minerals can be added to diets at lower concentrations without any adverse effects on birds’ growth performance while reducing mineral excretion compared to inorganic mineral sources [2,9]. The results of this study reconfirmed the benefits of MMHAC supplementation in reducing the excretion of Mn, Zn, and/or Cu compared with the normal levels (ITM) or high inorganic Cu levels (T125). Additionally, the current findings suggest that MMHAC supplementation at the higher Cu level (30 ppm) is more beneficial in lowering the excretion of other minerals including Mg, P, S, and Co than the lower Cu level (10 ppm) that might be due to the fewer antagonistic reactions between Cu and other minerals or other dietary components at this level. Interactions between Cu, Zn, and/or Mn during the feed digestion and absorption process have been documented in the literature [21,35]. Specifically, excessive supplemental Zn levels may induce Cu deficiency and vice versa as Cu and Zn bind to the same protein carriers in the mucosa leading to the competition between these minerals during the absorption process [35]. Others have indicated that Cu may also influence the accumulation of Mn in birds [36]. The current findings were consistent with those observed by Lee et al. [7] who reported lower excreta Cu and Zn levels in birds fed Cu-amino acid and Zn-amino acid chelates compared to those offered high levels of inorganic trace minerals. Furthermore, previous studies have shown that dietary Zn and Mn supplementation at 40 ppm and 60 ppm, respectively, as recommended by the NRC [37] was sufficient to support birds’ growth performance and bone health [21,38]. The results of this study and others [3] illustrated that the supplementation of inorganic Zn and Mn at high levels is wasteful as the birds’ bodies store these minerals to a very small extent.

The results of the current study and others [39] showed that the dietary inclusion of high inorganic Cu levels might improve birds’ growth performance at a young age but also result in excessive Cu excretion to the environment. The current findings were supported by Bao et al. [3] who reported that Cu digestibility was not affected by dietary Cu supplemental levels or intestinal segments of broilers. Similarly, Dozier III et al. [14] indicated that increasing dietary Cu supplemental levels increased the absolute amount of Cu excretion in birds; however, the relative Cu-excreted output to Cu intake was generally not different between the treatment groups. Additionally, Leeson [35] pointed out that, as the majority of supplemental Cu in the diets (at least 80%) is excreted in the excreta, lowering dietary Cu supplementation would reduce Cu end up in the environment. Skřivan et al. [40] observed that Cu levels in the excreta on a dry matter basis increased from 25 to 400 ppm when dietary inorganic Cu supplemental levels increased from 9 to 240 ppm. Other investigators reported that increasing Cu supplemental levels from 10 to 260 ppm as Cu sulphate increased Cu levels in the excreta from 27.5 to 281.9 ppm (an increase of 254.4 ppm) on a dry matter basis at day 42 [41]. In the current study, feeding 125 ppm of Cu as TBCC resulted in an increase of 257 ppm of Cu in the excreta on the dry matter basis (398 vs. 141 ppm) compared to the ITM treatment group with the dietary Cu level of 16 ppm as Cu sulphate at day 42. In contrast, a slight increase in Cu-excreted levels (25 ppm) was observed in birds fed the MMHAC diet at 30 ppm of Cu compared to those fed the ITM diet with 16 ppm of Cu as Cu sulphate at day 42 in the current study (166 vs. 141 ppm). Thus, by reducing excretion levels of Cu and other minerals, supplementing MMHACs at 30 ppm may be the more appropriate nutritional strategy to promote economic and environmental sustainability in broiler production compared to the high inorganic Cu diets. Furthermore, the practice of using high dietary Cu levels as a growth promotor in broiler production has been questioned as it may reduce the efficacy of phytase enzymes by decreasing the solubility of the phytic acid complex [35]. Additionally, previous studies have reported that high dietary Cu levels decreased P retention [42]. This finding is in good agreement with the results of the current study, which show that feeding 125 ppm of TBCC increased P levels in the birds’ excreta.

It was observed in the current study that birds offered the M10 and M30 diets had lower excreta Zn and Mn levels compared to those offered the T125 and ITM control diets while higher excreta Cu levels were noted in birds fed the T125 diet compared to those fed the other diets on all sampling days except day 35. The current findings also showed that birds fed the T125 diet had higher excreta Ca levels compared to the ITM diet on day 10 but lower excreta Ca levels compared to the ITM and M10 diets on day 28 and day 35 and lower excreta Ca levels compared to the M10 diet on day 42. These findings may reflect the effects of bird age and interactions between the minerals in the diet on the mineral excretion in broilers. It is known that mineral digestibility and absorption in birds are influenced by the bird age, dietary level of organic minerals, and feed intake [43]. Furthermore, Bao et al. [44] reported that the supplementation of either Cu, Zn, Mn, Fe, or combined Zn and Mn did not affect Ca excretion, while the combined supplementation of Cu, Zn, Mn, and Fe from either organic or inorganic sources decreased the Ca excretion of broilers from 21 to 24 days old. As mentioned above, decreased excreta Ca levels were also observed in birds fed the T125 diet (where high dietary Cu, Zn, and Mn levels were used) compared to the ITM and/or M10 diet on days 28, 35, and 42 in the current study. However, the mechanism of this effect is unclear. Previous studies have shown that increasing dietary Ca levels linearly reduced the Cu excretion of broilers on day 42 [45]. Cu and Ca may have a synergistic effect; however, this effect may depend on bird age and levels of other minerals in the diets, particularly Zn, Mn, and Fe.

Limited information could be found in the literature regarding the effects of feeding chelated trace minerals on environmental impacts, litter conditions, and air gas levels in broiler production. Manangi et al. [9] showed that feeding chelated Cu, Zn, and Mn at reduced levels significantly decreased litter trace mineral content while maintaining the growth performance of broiler chickens. Research in swine has shown that dietary chelated Cu and Zn inclusion did not affect the levels of fecal noxious gases including hydrogen sulphide, NH_3_, and total mercaptans [46,47]. It has been suggested that the fecal noxious gas level is mainly influenced by nitrogen digestibility as the microbial fermentation in the hind gut strongly depends on the amount of substrate passing from the foregut [48]. Ammonia has been considered a major product of the microbial fermentation of undigested nitrogen/protein in the birds’ ceca [49,50]. Hence, the similar levels of NH_3_ and other gases between the treatment groups in the current study might be associated with the similar nitrogen digestibility in the respective groups as reported by Nguyen et al. [24], whereas the similar excreta moisture content might be the reason for the similar litter conditions between the treatments in this study. However, the results of the current study showed that MMHAC supplementation at 30 ppm can be expected to improve the sustainability of the poultry industry in terms of reduced emissions into the environment, as simulated by the Poultry Greenhouse Accounting Framework V1.45 from the Primary Industries Climate Change Centre [33]. Emissions from poultry farms have raised public concerns in many parts of the world due to their possible harmful impacts on human health and animal welfare [51]. A 16-year data (from 1997 to 2013) showed that poultry production generates the highest emissions of odours [52]. Thus, enhancing birds’ productivity while minimizing environmental impacts is crucial to improve the economic and environmental sustainability of the broiler industry [16]. By comprehensively examining environmental aspects including resource utilization and the environmental consequences of activities occurring throughout the entire life cycle of a product, the life cycle assessment has been increasingly used to assess the environmental impacts of broiler production [17,18,19]. The current findings on the life cycle assessment demonstrated that the dietary supplementation of MMHACs at 30 ppm led to the lowest total CO2e/farm and total CO2e/farm per kilo live weight gain and, therefore, the highest improvement in environmental sustainability. The lower emission of the M30 treatment group compared to the other groups in the current study resulted from the improved weight gain and lower amount of emissions from CO_2_, CH_4_, and N_2_O (fuel, manure management, atmospheric deposition, leaching, and runoff), which may have a great implication for the broiler industry.

The presence of lesions on the foot pads may cause bacterial infections and have negative impacts on bird growth performance, carcass quality, health conditions, and welfare standards [53,54]. Both Cu and Zn play critical roles in supporting the integrity of skin and connective tissues and wound healing [55]. Additionally, it is known that Zn deficiency causes feet parakeratosis and delays wound healing [56] and Mn deficiency causes leg abnormalities [57]. Likewise, previous research demonstrates that the dietary supplementation of organic Zn decreased skin tearing and increased skin collagen content in broilers [23]. Zhao et al. [4] and Manangi et al. [9] reported the tendency of lower footpad lesion scores in birds fed diets supplemented with 40 ppm of Zn, 60 ppm of Mn, and 8 ppm of Cu from inorganic sources (sulphates) and 40 ppm of Zn, 60 ppm of Mn, and 8 ppm of Cu from chelated trace minerals (Mintrex) compared to those fed 100% inorganic trace minerals (sulphates) with 80 ppm of Zn, 120 ppm of Mn, and 8 ppm of Cu. The lack of MMHAC effects on foot pad lesion and hock burn scores may be associated with the lower Zn levels in the MMHAC diets in the current study compared to other studies. Furthermore, feeding MMHAC and T125 diets did not affect excreta and litter moisture content in the current study, which is one of the main factors affecting bird foot health. Meanwhile, the similar plumage cleanliness, leg deformity, and walking ability between the treatment groups in the current study might be attributed to the similar litter moisture content and bone parameters between the treatments (as reported by Nguyen et al. [24]). Nevertheless, the current findings illustrated that the supplementation of MMHACs with 40 ppm of Zn, 10 ppm of Cu, and 40 ppm of Mn was sufficient to maintain bird welfare conditions compared to the traditional diets with high ITM levels. Sunder et al. [58] demonstrated that dietary inorganic Zn supplementation at 29 ppm is sufficient to maintain normal hock joints and performance to 4 weeks of age. Similarly, Gajula et al. [21] reported that the dietary supplementation of inorganic Zn (40 to 160 ppm) and Mn (60 to 240 ppm) did not affect leg abnormality scores and tibia strength in broilers. These findings were consistent with the findings of the current study. However, 80 ppm from an inorganic source may be required to improve mineral retention and immune response and alleviate stress. In the current study, M10 and M30 diets were able to perform and reduce the excretion of Mn, Zn, and/or Cu compared with the normal levels (ITM) or high Cu levels (T125).

## 5. Conclusions

It can be concluded that the supplementation of MMHACs to broiler diets at 30 ppm is effective in maintaining litter quality and welfare status while reducing emissions into the environment and the Mn, Zn, and/or Cu excretion of broilers compared to diets supplemented with high levels of inorganic mineral sources, therefore reducing the environmental impacts of broiler production. The results of the current study may be used as a reference for broiler producers to calculate the reduction in environmental load when applying different mineral premixes to their production systems.

## Figures and Tables

**Table 1:** Excreta mineral content of broilers fed the dietary treatments at day 10.

Mineral	ITM	M10	T125	M30	SEM	*p*-Value
P, mg/g	7.64	8.06	8.01	7.93	0.16	0.345
Ca, mg/g	9.87 ^a^	12.34 ^bc^	13.87 ^c^	12.24 ^b^	0.41	<0.001
Mg, mg/g	5.46	5.48	5.44	5.45	0.09	0.993
K, mg/g	26.8	24.5	25.3	26.4	0.72	0.143
Na, mg/g	0.94 ^a^	1.26 ^bc^	1.52 ^c^	1.07 ^ab^	0.07	<0.001
S, mg/g	3.58	3.63	3.79	3.64	0.08	0.297
Cu, µg/g	96.7 ^ab^	59.4 ^a^	360.4 ^c^	141.9 ^b^	14.95	<0.001
Zn, µg/g	415 ^b^	286 ^a^	408 ^b^	259 ^a^	12.39	<0.001
Mn, µg/g	675 ^b^	575 ^a^	688 ^b^	542 ^a^	19.96	<0.001
Fe, µg/g	340	351	356	348	13.67	0.880
Al, µg/g	242	233	242	235	8.30	0.855
Cr, µg/g	1.68	1.66	1.65	1.61	0.06	0.860
Co, µg/g	0.767	0.715	0.728	0.680	0.020	0.081
B, µg/g	62.1	59.9	62.0	62.0	1.73	0.779

ITM: inorganic trace mineral ZnSO_4_ (110 ppm), CuSO_4_ (16 ppm), and MnO (120 ppm); M10: mineral methionine hydroxyl analogue chelates Zn (40 ppm), Cu (10 ppm), and Mn (40 ppm); T125: inorganic trace mineral ZnSO_4_ (110 ppm), tribasic copper chloride (125 ppm), and MnO (120 ppm); and M30: mineral methionine hydroxyl analogue chelates Zn (40 ppm), Cu (30 ppm), and Mn (40 ppm). ^a,b,c^ Differing superscripts within a row indicate significant differences between means. *p*-values ≤ 0.05 were considered significant.

**Table 2:** Excreta mineral content of broilers fed the dietary treatments at day 16.

Mineral	ITM	M10	T125	M30	SEM	*p*-Value
P, mg/g	9.51	9.11	9.00	9.21	0.40	0.832
Ca, mg/g	9.78	9.63	8.77	9.45	0.40	0.312
Mg, mg/g	6.58	6.21	6.24	6.13	0.12	0.060
K, mg/g	29.5	29.0	28.8	27.8	0.86	0.606
Na, mg/g	1.02	1.12	1.13	0.95	0.08	0.318
S, mg/g	4.27	4.26	4.32	3.94	0.17	0.421
Cu, µg/g	119 ^a^	130 ^a^	420 ^b^	129 ^a^	19.73	<0.001
Zn, µg/g	496 ^bc^	393 ^ab^	510 ^c^	295 ^a^	28.23	<0.001
Mn, µg/g	795 ^b^	615 ^a^	835 ^b^	569 ^a^	30.96	<0.001
Fe, µg/g	401	398	390	389	14.01	0.912
Al, µg/g	230	236	218	219	5.77	0.109
Cr, µg/g	1.59	1.56	1.99	1.47	0.26	0.538
Co, µg/g	0.952	0.817	0.838	0.823	0.04	0.109
B, µg/g	67.8	67.1	65.1	64.6	2.94	0.850

ITM: inorganic trace mineral ZnSO_4_ (110 ppm), CuSO_4_ (16 ppm), and MnO (120 ppm); M10: mineral methionine hydroxyl analogue chelates Zn (40 ppm), Cu (10 ppm), and Mn (40 ppm); T125: inorganic trace mineral ZnSO_4_ (110 ppm), tribasic copper chloride (125 ppm), and MnO (120 ppm); and M30: mineral methionine hydroxyl analogue chelates Zn (40 ppm), Cu (30 ppm), and Mn (40 ppm). ^a,b,c^ Differing superscripts within a row indicate significant differences between means. *p*-values ≤ 0.05 were considered significant.

**Table 3:** Excreta mineral content of broilers fed the dietary treatments at day 21.

Mineral	ITM	M10	T125	M30	SEM	*p*-Value
P, mg/g	9.16 ^ab^	9.16 ^ab^	9.60 ^b^	8.76 ^a^	0.18	0.050
Ca, mg/g	9.83	10.06	10.54	10.05	0.36	0.589
Mg, mg/g	5.88	5.87	5.99	5.79	0.12	0.708
K, mg/g	26.7	26.8	27.6	26.0	0.89	0.656
Na, mg/g	1.34	1.51	1.56	1.32	0.10	0.277
S, mg/g	3.91 ^b^	3.82 ^ab^	3.84 ^b^	3.51 ^a^	0.08	0.009
Cu, µg/g	72.4 ^a^	105 ^ab^	386 ^c^	134 ^b^	9.84	<0.001
Zn, µg/g	497 ^c^	319 ^b^	517 ^c^	257 ^a^	10.87	<0.001
Mn, µg/g	773 ^b^	546 ^a^	831 ^b^	490 ^a^	19.51	<0.001
Fe, µg/g	359	360	379	352	10.02	0.292
Al, µg/g	207	206	214	207	5.10	0.758
Cr, µg/g	1.77	1.72	1.86	1.66	0.06	0.132
Co, µg/g	0.905	0.889	0.835	0.752	0.041	0.066
B, µg/g	60.4	60.5	62.5	59.3	1.67	0.596

ITM: inorganic trace mineral ZnSO_4_ (110 ppm), CuSO_4_ (16 ppm), and MnO (120 ppm); M10: mineral methionine hydroxyl analogue chelates Zn (40 ppm), Cu (10 ppm), and Mn (40 ppm); T125: inorganic trace mineral ZnSO_4_ (110 ppm), tribasic copper chloride (125 ppm), and MnO (120 ppm); and M30: mineral methionine hydroxyl analogue chelates Zn (40 ppm), Cu (30 ppm), and Mn (40 ppm). ^a,b,c^ Differing superscripts within a row indicate significant differences between means. *p*-values ≤ 0.05 were considered significant.

**Table 4:** Excreta mineral content of broilers fed the dietary treatments at day 28.

Mineral	ITM	M10	T125	M30	SEM	*p*-Value
P, mg/g	8.35	8.43	8.22	8.09	0.37	0.926
Ca, mg/g	9.85 ^bc^	10.64 ^c^	7.91 ^a^	8.71 ^ab^	0.43	<0.001
Mg, mg/g	5.36	5.36	5.41	5.10	0.19	0.653
K, mg/g	25.7	27.3	28.4	26.9	1.15	0.441
Na, mg/g	2.34 ^b^	2.31 ^b^	1.72 ^a^	1.77 ^ab^	0.14	0.008
S, mg/g	3.41 ^b^	3.21 ^ab^	3.37 ^b^	3.02 ^a^	0.08	0.018
Cu, µg/g	70.5 ^a^	59.1 ^a^	428.7 ^c^	134.3 ^b^	8.02	<0.001
Zn, µg/g	506 ^b^	238 ^a^	464 ^b^	225 ^a^	14.40	<0.001
Mn, µg/g	725 ^b^	443 ^a^	712 ^b^	406 ^a^	22.91	<0.001
Fe, µg/g	297	317	312	295	10.99	0.462
Al, µg/g	179	176	173	165	6.15	0.429
Cr, µg/g	1.21	1.18	1.16	1.15	0.05	0.874
Co, µg/g	0.697 ^b^	0.481 ^a^	0.612 ^ab^	0.451 ^a^	0.042	0.001
B, µg/g	53.5	54.9	54.1	52.2	1.79	0.756

ITM: inorganic trace mineral ZnSO_4_ (110 ppm), CuSO_4_ (16 ppm), and MnO (120 ppm); M10: mineral methionine hydroxyl analogue chelates Zn (40 ppm), Cu (10 ppm), and Mn (40 ppm); T125: inorganic trace mineral ZnSO_4_ (110 ppm), tribasic copper chloride (125 ppm), and MnO (120 ppm); and M30: mineral methionine hydroxyl analogue chelates Zn (40 ppm), Cu (30 ppm), and Mn (40 ppm). ^a,b,c^ Differing superscripts within a row indicate significant differences between means. *p*-values ≤ 0.05 were considered significant.

**Table 5:** Excreta mineral content of broilers fed the dietary treatments at day 35.

Mineral	ITM	M10	T125	M30	SEM	*p*-Value
P, mg/g	8.20	8.46	7.76	7.26	0.36	0.123
Ca, mg/g	10.19 ^b^	10.22 ^b^	7.83 ^a^	9.00 ^ab^	0.44	0.003
Mg, mg/g	4.79 ^c^	4.62 ^bc^	4.16 ^a^	4.34 ^ab^	0.10	0.002
K, mg/g	27.6	29.0	29.1	26.7	1.08	0.351
Na, mg/g	3.05	2.88	2.48	2.72	0.21	0.296
S, mg/g	3.85	3.81	3.81	3.65	0.11	0.607
Cu, µg/g	131	115	236	142	42.24	0.209
Zn, µg/g	377	295	261	258	37.38	0.112
Mn, µg/g	612	540	498	450	55.10	0.226
Fe, µg/g	311 ^b^	297 ^ab^	268 ^a^	273 ^a^	8.97	0.009
Al, µg/g	137	137	127	120	7.89	0.417
Cr, µg/g	1.42	1.30	1.26	1.20	0.06	0.091
Co, µg/g	0.625 ^b^	0.521 ^ab^	0.482 ^a^	0.483 ^a^	0.034	0.024
B, µg/g	49.8	50.9	48.0	47.1	1.58	0.343

ITM: inorganic trace mineral ZnSO_4_ (110 ppm), CuSO_4_ (16 ppm), and MnO (120 ppm); M10: mineral methionine hydroxyl analogue chelates Zn (40 ppm), Cu (10 ppm), and Mn (40 ppm); T125: inorganic trace mineral ZnSO_4_ (110 ppm), tribasic copper chloride (125 ppm), and MnO (120 ppm); and M30: mineral methionine hydroxyl analogue chelates Zn (40 ppm), Cu (30 ppm), and Mn 40 ppm. ^a,b,c^ Differing superscripts within a row indicate significant differences between means. *p*-values ≤ 0.05 were considered significant.

**Table 6:** Excreta mineral content of broilers fed the dietary treatments at day 42.

Mineral	ITM	M10	T125	M30	SEM	*p*-Value
P, mg/g	9.87	9.71	9.70	10.49	0.39	0.532
Ca, mg/g	11.41 ^ab^	11.67 ^b^	9.53 ^a^	10.74 ^ab^	0.52	0.037
Mg, mg/g	5.96	5.85	5.88	5.94	0.16	0.969
K, mg/g	28.1	26.1	27.8	27.8	1.03	0.589
Na, mg/g	3.19	2.85	2.84	3.01	0.20	0.638
S, mg/g	4.74	4.46	4.32	4.30	0.15	0.200
Cu, µg/g	141 ^a^	131 ^a^	398 ^b^	166 ^a^	33.55	<0.001
Zn, µg/g	470 ^b^	339 ^a^	429 ^ab^	330 ^a^	25.08	0.002
Mn, µg/g	701 ^b^	518 ^a^	670 ^b^	518 ^a^	31.46	<0.001
Fe, µg/g	366	389	386	387	11.31	0.503
Al, µg/g	194	198	182	194	9.54	0.652
Cr, µg/g	1.67	1.60	1.64	1.63	0.07	0.897
Co, µg/g	0.857	0.771	0.811	0.669	0.069	0.387
B, µg/g	55.8	54.5	57.0	58.7	1.53	0.342

ITM: inorganic trace mineral ZnSO_4_ (110 ppm), CuSO_4_ (16 ppm), and MnO (120 ppm); M10: mineral methionine hydroxyl analogue chelates Zn (40 ppm), Cu (10 ppm), and Mn (40 ppm); T125: inorganic trace mineral ZnSO_4_ (110 ppm), tribasic copper chloride (125 ppm), and MnO (120 ppm); and M30: mineral methionine hydroxyl analogue chelates Zn (40 ppm), Cu (30 ppm), and Mn (40 ppm). ^a,b^ Differing superscripts within a row indicate significant differences between means. *p*-values ≤ 0.05 were considered significant.

**Table 7:** Excreta nitrogen content of broilers fed the dietary treatments (as per dry matter basis, %).

Bird Age (Days Old)	ITM	M10	T125	M30	SEM	*p*-Value
10	4.06 ^c^	3.86 ^ab^	3.75 ^a^	3.98 ^bc^	0.050	<0.001
16	3.96	4.10	4.06	4.09	0.057	0.308
21	4.07	4.03	4.19	3.98	0.127	0.711
28	4.21	4.26	4.40	4.35	0.087	0.544
35	4.78	5.02	5.11	4.87	0.132	0.325
42	4.89	4.91	4.72	4.57	0.175	0.515

ITM: inorganic trace mineral ZnSO_4_ (110 ppm), CuSO_4_ (16 ppm), and MnO (120 ppm); M10: mineral methionine hydroxyl analogue chelates Zn (40 ppm), Cu (10 ppm), and Mn (40 ppm); T125: inorganic trace mineral ZnSO_4_ (110 ppm), tribasic copper chloride (125 ppm), and MnO (120 ppm); and M30: mineral methionine hydroxyl analogue chelates Zn (40 ppm), Cu (30 ppm), and Mn (40 ppm). ^a,b,c^ Differing superscripts within a row indicate significant differences between means. *p*-values ≤ 0.05 were considered significant.

**Table 8:** Excreta and litter moisture content and litter score of broilers fed the dietary treatments.

Variable	ITM	M10	T125	M30	SEM	*p*-Value
Excreta moisture day 10 (%)	80.2	80.5	80.5	80.8	0.23	0.416
Excreta moisture day 16 (%)	81.1	81.5	80.9	81.5	0.34	0.500
Excreta moisture day 21 (%)	80.7	80.4	81.4	81.0	0.31	0.193
Litter moisture day 21 (%)	40.3	40.0	42.0	43.3	2.67	0.809
Litter score day 21	1.15	1.10	1.00	1.10	0.03	0.056
Excreta moisture day 28 (%)	81.1	81.2	80.5	80.6	0.36	0.411
Excreta moisture day 35 (%)	81.3	80.1	80.5	80.2	0.39	0.185
Excreta moisture day 42 (%)	80.5	80.1	80.2	80.6	0.36	0.738
Litter moisture day 42 (%)	28.8	26.0	26.7	26.6	1.12	0.366
Litter score day 42	0.525	0.475	0.425	0.300	0.140	0.493

ITM: inorganic trace mineral ZnSO_4_ (110 ppm), CuSO_4_ (16 ppm), and MnO (120 ppm); M10: mineral methionine hydroxyl analogue chelates Zn (40 ppm), Cu (10 ppm), and Mn (40 ppm); T125: inorganic trace mineral ZnSO_4_ (110 ppm), tribasic copper chloride (125 ppm), and MnO (120 ppm); and M30: mineral methionine hydroxyl analogue chelates Zn (40 ppm), Cu (30 ppm), and Mn (40 ppm).

**Table 9:** Ammonia, carbon dioxide, and methane levels at the broiler shed at days 21 and 42 (ppm/bird).

Air Gas Level (ppm/bird)		ITM	M10	T125	M30	SEM	*p*-Value
Day 21	Ammonia	0.068	0.074	0.042	0.091	0.018	0.321
	Carbon dioxide	149	150	152	151	3.951	0.945
Day 42	Ammonia	0.763	0.768	0.825	0.850	0.098	0.903
	Carbon dioxide	123	103	123	125	7.551	0.164
	Methane	0.137	0.174	0.195	0.160	0.083	0.968

ITM: inorganic trace mineral ZnSO_4_ (110 ppm), CuSO_4_ (16 ppm), and MnO (120 ppm); M10: mineral methionine hydroxyl analogue chelates Zn (40 ppm), Cu (10 ppm), and Mn (40 ppm); T125: inorganic trace mineral ZnSO_4_ (110 ppm), tribasic copper chloride (125 ppm), and MnO (120 ppm); and M30: mineral methionine hydroxyl analogue chelates Zn (40 ppm), Cu (30 ppm), and Mn 40 ppm.

**Table 10:** Simulated emissions (CO_2_; CH_4_; N_2_O), the total t CO2e/farm and the total t CO2e/farm per kilo live weight gain.

Variable	ITM	M10	T125	M30	SEM	*p*-Value
Emissions; CO_2_; CH_4_; N_2_O (fuel, manure management, atmospheric deposition, leaching and runoff) (t CO2e)	6985 ^a^	7394 ^b^	7331 ^ab^	6935 ^a^	61.92	0.007
Emissions, purchased feed, electricity, and fuel (t CO2e)	2190	2202	2229	2219	5.82	0.076
Total t CO2e/farm (t CO2e)	10,486 ^ab^	10,906 ^b^	10,870 ^ab^	10,464 ^a^	66.28	0.013
Total t CO2e/farm per kilo live weight gain (t CO2e)	0.01484 ^a^	0.01481 ^a^	0.01547 ^b^	0.01431 ^a^	0.00010	<0.001

CO_2_: carbon dioxide; CH_4_: methane, N_2_O: nitrous oxide; ITM: inorganic trace mineral ZnSO_4_ (110 ppm), CuSO_4_ (16 ppm), and MnO (120 ppm); M10: mineral methionine hydroxyl analogue chelates Zn (40 ppm), Cu (10 ppm), and Mn (40 ppm); T125: inorganic trace mineral ZnSO_4_ (110 ppm), tribasic copper chloride (125 ppm), and MnO (120 ppm); and M30: mineral methionine hydroxyl analogue chelates Zn (40 ppm), Cu (30 ppm), and Mn 40 ppm. ^a,b^ Differing superscripts within a row indicate significant differences between means. *p*-values ≤ 0.05 were considered significant.

**Table 11:** Welfare indicators of broilers fed the dietary treatments at days 21 and 42.

Welfare Indicator Score		ITM	M10	T125	M30	SEM	*p*-Value
Day 21	Foot pad lesion	0.042	0.000	0.000	0.021	0.012	0.270
	Plumage cleanliness	0.333	0.167	0.333	0.250	0.091	0.541
	Walking ability	0.208	0.167	0.250	0.208	0.083	0.927
Day 42	Foot pad lesion	0.042	0.000	0.000	0.000	0.008	0.103
	Hock burn	0.385	0.396	0.271	0.271	0.078	0.519
	Plumage cleanliness	1.833	2.083	2.042	1.792	0.117	0.229
	Leg deformity	0.083	0.083	0.042	0.000	0.038	0.471
	Walking ability	2.250	1.833	2.000	1.792	0.151	0.163

ITM: inorganic trace mineral ZnSO_4_ (110 ppm), CuSO_4_ (16 ppm), and MnO (120 ppm); M10: mineral methionine hydroxyl analogue chelates Zn (40 ppm), Cu (10 ppm), and Mn (40 ppm); T125: inorganic trace mineral ZnSO_4_ (110 ppm), tribasic copper chloride (125 ppm), and MnO (120 ppm); and M30: mineral methionine hydroxyl analogue chelates Zn (40 ppm), Cu (30 ppm), and Mn (40 ppm).

## Data Availability

The data that support this study will be shared upon reasonable request with the corresponding author.

## Appendix A

**Table A1:** Diet composition and calculated nutrient values of the basal/control diets (as-fed basis).

Ingredients, %	Starter (Days 0–10)	Grower (Days 10–21)	Finisher (Days 21–42)
Wheat	38.89	34.37	38.94
Sorghum	20.00	30.00	30.00
Soybean meal	34.33	29.09	25.65
Canola oil	2.84	2.92	2.83
Limestone	1.14	0.86	0.79
Dicalcium phosphate	1.13	0.75	0.45
Xylanase (Econase XT 5P)	0.002	0.002	0.002
Phytase (Quantum Blue 5G)	0.01	0.01	0.01
Salt	0.20	0.17	0.19
Sodium bicarbonate	0.23	0.17	0.16
Titanium dioxide	0.00	0.50	0.00
Vitamin-mineral premix ^1^	0.10	0.10	0.10
Choline Cl 60%	0.048	0.054	0.041
L-lysine HCl	0.362	0.341	0.309
D,L-methionine	0.438	0.395	0.351
L-threonine	0.176	0.149	0.118
L-arginine	0.050	0.067	0.056
L-valine	0.056	0.044	0.023
Calculated nutrient, % (otherwise as stated)			
Dry matter	91.0	90.9	90.9
AMEn ^2^, kcal/kg	2975	3050	3100
Crude protein	22.9	20.9	19.7
Crude fat	4.76	5.05	5.02
Crude fibre	3.20	3.42	3.39
Ash content	4.68	4.47	3.50
Dig. ^3^ lysine	1.320	1.180	1.080
Dig. methionine	0.708	0.647	0.592
Dig. methionine + cysteine	1.000	0.920	0.860
Dig. threonine	0.880	0.790	0.720
Calcium	0.950	0.750	0.650
Available phosphorus	0.500	0.420	0.360
Sodium	0.210	0.180	0.180
Potassium	1.013	0.918	0.863
Chloride	0.250	0.230	0.230
Choline, mg/kg	1700	1600	1500
Linoleic acid	1.686	1.803	1.813
Dietary electrolyte balance, mEq/kg	302	269	253

^1^ Vitamin–mineral premixes used in the experimental diets prepared and provided by an experienced manufacturer (Rabar Pty Ltd., Beaudesert, Queensland, Australia). The vitamin–mineral premix in the basal/control diet was replaced with the respective vitamin–mineral premix for each treatment to generate M10, T125, and M30 diets. Levels of supplemental D,L-methionine were reduced in the M10 and M30 diets to generate similar methionine levels across the treatments; ^2^ AMEn: apparent metabolizable energy corrected to zero N retention; ^3^ Dig: standard ileal digestible amino acid coefficients as determined by Near-Infra Red spectroscopy (Foss NIR 6500, Denmark) standardized with Adisseo calibration.

**Table A2:** Analyzed nutrient values of experimental diets (as-fed basis).

Nutrients	Starter (Days 0–10)				Grower (Days 10–21)				Finisher (Days 21–42)			
	ITM	M10	T125	M30	ITM	M10	T125	M30	ITM	M10	T125	M30
DM, %	89.3	88.6	89.5	89.2	89.5	89.7	90.3	89.0	89.7	89.6	89.6	89.7
GE, kcal/kg	4039	3997	4025	4021	4038	4046	4079	4000	4074	4082	4083	4084
CP, %	22.0	22.0	22.8	22.0	20.8	21.0	21.5	21.2	19.5	19.3	19.5	19.8
Cu, µg/g	19.8	20.2	123.8	55.1	21.7	20.5	135.5	45.8	18.2	20.9	137.2	44.8
Zn, µg/g	115.2	71.9	117.6	72.2	124.2	68.1	119.6	60.9	123.1	57.9	122.9	58.9
Mn, µg/g	166	137	167	139	203	121	192	117	172	111	171	105
P, %	0.66	0.67	0.66	0.66	0.60	0.59	0.59	0.57	0.51	0.52	0.51	0.50
Ca, %	0.74	0.84	0.85	0.81	0.64	0.68	0.63	0.60	0.57	0.58	0.52	0.52
Mg, %	0.19	0.19	0.18	0.18	0.18	0.18	0.18	0.18	0.17	0.17	0.17	0.16
Na, %	0.13	0.15	0.15	0.14	0.12	0.13	0.12	0.12	0.13	0.14	0.12	0.12
K, %	1.13	1.09	1.07	1.08	1.00	0.99	1.00	0.99	0.92	0.92	0.91	0.90
S, %	0.32	0.33	0.34	0.32	0.31	0.30	0.31	0.29	0.30	0.29	0.28	0.28
Al, µg/g	68.5	71.8	71.6	65.6	52.7	53.2	53.6	49.6	51.9	47.8	43.9	44.5
Fe, µg/g	99.5	114.3	108.9	102.0	100.3	98.1	99.6	89.8	89.2	90.4	86.7	88.1
B, µg/g	17.0	16.5	15.9	15.9	15.5	15.4	15.6	15.4	13.8	13.7	12.5	11.9
Cr, µg/g	0.38	0.46	0.40	0.39	1.24	1.15	1.23	1.06	0.57	0.58	0.34	0.27
Co, µg/g	2.24	1.07	0.90	0.67	0.91	0.69	0.87	0.61	0.78	0.65	0.65	0.50

ITM: inorganic trace mineral ZnSO_4_ (110 ppm), CuSO_4_ (16 ppm), and MnO (120 ppm); M10: mineral methionine hydroxyl analogue chelates Zn (40 ppm), Cu (10 ppm), and Mn (40 ppm); T125: inorganic trace mineral ZnSO_4_ (110 ppm), tribasic copper chloride (125 ppm), and MnO (120 ppm); M30: mineral methionine hydroxyl analogue chelates Zn (40 ppm), Cu (30 ppm), and Mn (40 ppm); DM: dry matter; GE: gross energy; and CP: crude protein.
